# Case Report: Serial Cases: Prolongation of High Immunoglobulin G Level in Repetitive COVID-19 Convalescence Plasma Donor in Saiful Anwar Hospital Malang, Indonesia

**DOI:** 10.3389/fimmu.2021.633323

**Published:** 2021-11-01

**Authors:** Nina Nurarifah, Herwindo Pudjo Brahmantyo, Shinta Oktya Wardhani, Djoko Heri Hermanto, Putu Moda Arsana

**Affiliations:** ^1^ Hematology and Medical Oncology Division, Department of Internal Medicine, Dr. Saiful Anwar Hospital, Malang, Indonesia; ^2^ Endocrine, Metabolid, and Diabetes Division, Department of Internal Medicine, Dr. Saiful Anwar Hospital, Malang, Indonesia

**Keywords:** Convalescent plasma, repetitive donor, COVID-19, immunoglobulin G, antibody

## Abstract

**Background:**

Convalescent plasma therapy is expected to be a promising alternative to supportive therapy during the SARS-CoV-2 pandemic outbreak. Altered immune response in repetitive convalescent plasma donors has not been widely studied. This case series was reported to analyze the patterns of immune responses and the factors that might influence them in repetitive convalescent plasma donors and increase awareness of COVID-19 survivors to donate their convalescent plasma.

**Cases Illustration:**

There were five repetitive donors who were eligible as convalescent plasma donor requirements. It was found two donors who showed increment of anti-SARS-CoV-2 IgG level after donation and two others who showed persistent anti-SARS-CoV-2 IgG level more than two months after recovered.

**Discussion:**

There was a difference in immune response in survivors who have the probability of being exposed to same antigens with survivors who did not, where the group of survivors who are at risk of exposure to antigens after recovery could trigger anamnestic immune response that can increase antiSARS-CoV-2 IgG levels. The other factor that influence the prolongation of anti-SARS-CoV-2 IgG levels are the possibility of neutralizing antibodies in plasma upregulation.

**Conclusion:**

Immunological phenomenon in SARS-CoV-2, both in survivors and convalescent plasma donors, have not been widely observed and studied. From the case series discussed above, it can be concluded that convalescent plasma donation does not yet have strong evidence of decreasing levels of specific antibodies against SARS-CoV-2 and plasmapheresis procedure is safe to be done without reducing the protective effect of donor antibody post-plasma donation.

## Background

Coronavirus disease 2019 (COVID-19) is a pandemic in which no particular effective therapeutic agent has been declared. The discovery of the right method of treatment is very important. Convalescence plasma transfusions are methods in which plasma containing antibodies is collected from donors who have recovered from COVID-19. It is based on past experience that shows that convalesence plasma therapy is quite effective and shows a good outcome (1).

Convalescent plasma had been used to treat outbreaks of other infections in the past. Convalescent plasma was used against the corona virus in the Severe Acute Respiratory Syndrome 1 (SARS-COV-1) outbreak in 2003, with satisfactory results. Compared with the control group, patients who received convalescent plasma had significantly faster mean time to hospital discharge (77.8% *vs* 23%; p = 0.004) and lower mortality rates (0% *vs* 23.8%; p = 0.049) (2). Convalescent plasma was also used in the Ebola epidemic outbreak in South Africa in 2013. About 84 patients received 500 mL of convalescent plasma. Compared with the control group, patients who received convalescent plasma had a shorter duration of symptoms (3). Based on previous experience, convalescent plasma therapy is expected to be a promising alternative to supportive therapy during the SARS-CoV-2 pandemic outbreak.

There are some obstacles that arise in raising convalescence plasma donors. Among them are concerns about declining levels of antibodies after donation. Since the beginning of COVID-19 pandemic, research about the benefits of convalescence plasma therapy for COVID-19 patients with critical condition has been widely done and published. However, the altered immune response in repetitive convalescent plasma donors has not been widely studied. This case series was written to analyze the patterns of convalescent plasma donor immune responses and the factors that may influence them. The purpose of this case series is increasing knowledge about the regulation of the immune response in repetitive convalescent plasma donors and increase awareness of COVID-19 survivors to donate their convalescent plasma.

## Cases Illustration

This case series reports five convalescent plasma donors who had met the convalescent plasma donor requirements: 18-60 years of age, had been declared cured of COVID-19 and had been symptom-free for at least 14 days, do not have infectious diseases through blood transfusions (Hepatitis B, Hepatitis C, syphilis, and HIV), do not have serious comorbid diseases (uncontrolled hypertension and diabetes mellitus, kidney failure, cancer, or heart failure), anti-SARS-CoV-2 IgG titer showed (at least) ≥ 1: 320, and RT-PCR results from naso-oropharyngeal swabs prior to plasmapheresis were negative. These requirements were taken from protocol study of Efficacy and safety of convalescent plasma transfusion administered as adjunctive treatment to standard treatment in moderate, severe, and/or critically ill patients with COVID-19 by Ministry of Research and Technology/National Research and Innovation Agency. The examination of anti-SARS-CoV-2 IgG levels was performed using a lateral flow ICT Biosensor from South Korea. Plasmapheresis was performed using the Haemonetics^®^ machine: MCS^®^ + Mobile Collection System 09000-220-EW at the Blood Transfusion Unit at dr. Saiful Anwar Hospital, Malang. All screening and plasmapheresis processes were performed at dr. Saiful Anwar Hospital, Malang and all of donors had signed the informed consent in front of the conventional plasma team of dr. Saiful Anwar Hospital, Malang.

As shown in **Table 1**, four plasma donors were male, and one plasma donor were female (ARI), where the donor ARI had never been pregnant before. Three donors had BMIs above normal, one donor had comorbid hypertension that was under controlled by routine medications, while one donor had no comorbidities.

**Table 1:** Epidemiology of repetitive convalescent plasma donors.

Donor Code	Sex	Age (years)	Occupation	Pneumonia	Comorbid
ARI	F	40	Healthcare provider	N	*Overweight*
FAN	M	34	Employee	Y	Obesity
SON	M	31	Healthcare provider	Y	None
RAC	M	48	Employee	Y	Hypertension
YOG	M	34	Healthcare provider	Y	Obesity

N, No; Y, Ya; M, Male; F, Female.

Three donors are healthcare providers (SON, ARI, and YOG) who are in charged in COVID ward. Both of them are suspected of having had the transmission from a patient that being treated with COVID. They handled COVID-19 patients at the beginning or the first cases in Malang were found, where PPE and the patient flow system as an adjustment to the COVID pandemic had not been properly developed.

The other two repetitive plasma donor worked as employees. RAC is an online motorcycle taxi driver who still routinely worked during the pandemic, while FAN is a production employee at a factory and is probably infected by colleagues who were also experiencing the same illness.

**Table 2** showed the symptoms experienced by our five repetitive donors when they were initially confirmed positive for COVID-19. Five of them had fever, cough, and breathlessness. Two persons experienced symptoms of diarrhea, while another person experienced anosmia. SON and YOG needed ventilation support when they were hospitalized (NIV), while FAN and RAC wore non-rebreathing mask support. Four of the repetitive donors hospitalized for 10-21 days. They were discharged after RT-PCR results from naso-oropharyngeal swab showed negative.

**Table 2:** Clinical features of repetitive convalescent plasma donors.

Donor Code	Symptoms					Ventilation support in hospitalization	Period of Hospitalization
	Diarrhea	Fever	Cough	Anosmia	Breathlessness		
**ARI**	+	+	+	–	+	No	Not hospitalized
**FAN**	–	+	+	–	+	No	21 days
**SON**	–	+	+	–	+	Yes	18 days
**RAC**	–	+	+	+	+	No	10 days
**YOG**	+	+	+	–	+	Yes	14 days

+ : symptom donor had while was in COVID-19 infection.

- : symptom donor did not had while was in COVID-19 infection.

These five plasma donors have donated more than once with a gap of at least 14 days (from the previous donation). Re-screening was performed according to requirements, including examination of anti-SARS-CoV-2 IgG levels and naso-oropharyngeal RT PCR swab prior plasmapheresis. Among the five plasma donors, two donors (ARI and SON) had showed elevation of anti-SARS-CoV-2 IgG levels at the 2^nd^ and 3^th^ plasma donations (1: 640) (**Table 3**). ARI showed persistent anti-SARS-CoV-2 IgG levels (1:320) until 96 days after recovered. SON showed another anti-SARS-CoV-2 IgG levels increment at the 4^th^ plasma donation that had been done 113 days after he recovered. FAN and RAC showed persistent anti-SARS-CoV-2 IgG levels (1:320) until 81 and 30 days (respectively) after recovered. None of donor experienced adverse events during and/or after convalescent plasma donation.

**Table 3:** Comparison of titration days and anti-SARS-CoV-2 IgG levels during preparation for convalescent plasma donation.

Donor Code	Blood Type	Anti-SARS-CoV-2 IgG Titration Day		Anti-SARS-CoV-2 IgG Levels	Plasma obtained (mL)	Adverse reactions
		Number of plasma donation	Days after recovered			
ARI	A	1	28	1:320	600	None
		2	49	1:640	600	
		3	71	1:320	200	
		4	96	1:320	200	
FAN	B	1	18	1:320	800	None
		2	38	1:320	800	
		3	81	1:320	800	
SON	AB	1	16	1:320	800	None
		2	33	1:320	800	
		3	88	1:640	800	
		4	113	1:1280	800	
RAC	B	1	14	1:320	600	None
		2	30	1:320	600	
YOG	O	1	43	1:320	800	None
		2	89	1:320	800	

Prior initiating convalescent plasma donation, all donors were examined the baseline laboratory. Five convalescent plasma donors showed different changes in lymphocyte levels. ARI showed a decrease in lymphocyte levels at each plasma donation, whereas FAN and SON showed a fluctuating lymphocyte level, and RAC showed an increase in lymphocyte levels. Fluctuation in albumin levels was also observed in the five donors, where SON and RAC showed relatively lower levels of albumin than the previous donation, while ARI and FAN showed fluctuating albumin levels (**Table 4**).

**Table 4:** Laboratory results at convalescent plasma donation screening.

Donor Code	Hb (gr/dL)	Leucocyte (/uL)	Thrombocyte (/uL)	Absolute neutrophil	Absolute lymphocyte	Albumin (gr/dL)
**ARI**	12.9	5,360	321,000	1,690	2,850	4.30
	13.90	6,940	347,000	3,330	2,670	4.04
	13.6	7,320	285,000	3,600	2,740	4.22
	12.50	6,500	320,000	3,200	2,490	4.17
**FAN**	15.20	7,040	304,000	3,770	2,410	4.72
	14.80	6,520	281,000	3,720	1,900	4.80
	14.9	7,760	281,000	4,500	2,280	4.64
**SON**	16.20	6,040	273,000	4,000	2,500	4.93
	15.6	7,060	242,000	4,080	2,300	4.96
	16.0	8,940	255,000	5,490	2,610	4.75
	16.1	7,390	249,000	4,330	2,370	4.74
**RAC**	11.8	6,250	246,000	4,120	1,440	4.58
	12.9	8,750	316,000	5,840	1,950	4.36
**YOG**	15.4	10,970	351,000	7,200	2,570	4,48
	15,7	9,920	430,000	5,210	3,810	4,80

## Discussion

Convalescent plasma had been used to treat outbreaks of other infections in the past. Convalescent plasma was used against the corona virus in the Severe Acute Respiratory Syndrome 1 (SARS-COV-1) outbreak in 2003, with satisfactory results. Based on previous experience, convalescent plasma therapy is expected to be a promising alternative to supportive therapy during the SARS-CoV-2 pandemic outbreak.

Determination of convalescent plasma quality against COVID-19 cannot be separated from the concept of immunology and the factors that influence it. Limited information as well as accurate research on immunological changes in SARS-CoV-2 infection means that knowledge about COVID-19 convalescent plasma is limited to hypotheses and relationships with pre-existing theories.

Plasma components that play an important role in determining the quality of convalescent plasma are the levels of neutralizing antibodies. In addition to increasing viral clearance, neutralizing antibodies can also reduce disease severity and aid patient recovery by modulating excessive immune responses (cytokine storms) associated with the severity of multi-organ failure (4–7). The FDA recommends a neutralizing antibody titer of at least 1: 160, but a titer of 1:80 is still acceptable if a suitable alternative unit is not available. However, assays to determine neutralizing antibody titres are not widely available because they require sample human resources and a level 3 biosafety laboratory if live viruses are used (8). This is also an obstacle in dr. Saiful Anwar hospital Malang. Thus, an enzyme-linked immunosorbent assay-based anti-SARS-CoV-2 IgG antibody titer was used as a parameter for donor antibody levels, which are considered to be correlated with neutralizing antibody titers (9).

IgG levels are lowest in the early stages of disease but slowly increase 15 days after the onset of symptoms. IgG then reaches a peak for 21-25 days and is maintained high for 31-41 days, during which time IgG is a parameter that can be used as a diagnostic tool in advanced/past infectious diseases (10). In this study, anti-SARS-CoV-2 IgG levels of all convalescent plasma donors were titrated on day 14 post-symptom-free and a result was at least ≥ 1: 320.

An interesting phenomenon were found in two convalescent plasma donors who showed increased anti-SARS-CoV-2 IgG titers after the previous plasma donation, namely ARI (on the second plasma donation) and SON (on the third and fourth plasma donation). The hypothesis that can be put forward is the possibility of anamnestic response in these two donors (as their profession as healthcare providers who involved in treating COVID-19 patients), where the probability of exposure to the same antigen can occur. Anamnestic response or secondary immune response is an immune response that occurs with the second and subsequent exposure to an antigen. Compared with the primary immune response, the anamnestic response has a shorter lag period with much smaller doses of antigen required to initiate these responses, but antibody titres peak higher and last longer. The anamnestic response is dominated by the production of IgG with a higher affinity for antigen (6). In a study by Chen et al. (11), there were 105 medical personnel who showed seropositive immunoassay results after they had contact with 4 confirmed cases of COVID-19, but the PCR swab results remained negative. This suggests that there is a possibility of asymptomatic or subclinical exposure to SARS-CoV-212. Based on this study, a hypothesis can be made regarding the possibility of re-exposure of SARS-CoV-2 in the workplace of both donors which could trigger an anamnestic response so that the anti-SARS-CoV-2 IgG titer increases even though it has exceeded three months post-recovery (12).

Different results were obtained in two other donors who were not healthcare providers, both of which (FAN and RAC) showed consistent anti-SARS-CoV-2 IgG titers (1: 320) during the convalescent plasma donation process. Some survivors’ concern about a decrease in post-donation plasma antibody levels has not been proven, but based on a study conducted by Gudbjartsson et al. (13) in Iceland, 91.1% of people showed seropositive antiviral antibody titers that increased within 2 months after being confirmed with COVID- 19 by qPCR and maintained high for up to 4 months (13). However, Li G et al. (14) stated that persistent IgG production can indicate IgG activity in the humoral immune response to acute SARS-CoV-2 infection and viral clearance remaining during recovery, so it is suggested that screening of COVID-19 convalescent plasma is necessary, including ensuring that the donor is free of SARS-CoV-2 by confirming negative for the PCR swab test (14).

There is no clear and scientifically proven upregulation mechanism related to plasma and/or plasma donor convalescent COVID-19. However, Winter JL (2006) stated in his journal of apheresis donor complications, frequent donors demonstrated a more sustained rise in reticulated platelets but the level attained was significantly less than that seen first time donors (15). This could be a new hypothesis that needs to be proven whether there is an upregulation in convalescent plasma donors that triggers plasma regeneration of younger and better quality than before so that antibody levels can be maintained or increased along with the frequency of plasma donors.

The five convalescent plasma donors showed different changes in lymphocyte levels. ARI showed a decrease in lymphocyte levels at each plasma donation, whereas FAN and SON showed a fluctuating lymphocyte level, and RAC showed an increase in lymphocyte levels. More recent studies of donors donating with newer hemapheresis instruments have shown no diffierences in lymphocyte counts, lymphocyte subsets, or IgG levels when comparing non-donors, plateletpheresis donors, and whole blood donors (16).

Fluctuation in albumin levels was also observed in the five donors, where SON and RAC showed relatively lower levels of albumin than the previous donation, while ARI and FAN showed fluctuating albumin levels. With plasma donations, similar concerns have been expressed with regard to inducing deficiency in humoral immunity. Theoretically, depletion of immunoglobulins faster than they can be replenished could occur, especially in frequent donors. Studies of long-term donors as well as donors undergoing frequent donations have not supported these concerns (17, 18).

Plasmapheresis is one of the methods of extracorporeal blood purification involving the removal of inflammatory mediators and antibodies (15). Szczeklik et al. (19) made a study involved 370 plasmapheresis procedures in 54 patients. It was observed that the most frequent adverse side effects during plasma filtration were decreases in arterial blood pressure (8.4% of all procedures), arrhythmias (3.5%), sensations of cold with temporarily elevated temperature and paresthesias (1.1%, each). In most cases, the symptoms were mild and transient. Severe and life-threatening episodes, i.e. shock, drops in arterial blood pressure requiring catecholamines administration, persistent arrhythmias and hemolysis, developed in 2.16% of procedures (19). These serial cases showed there was none of the donors reported adverse events during and/or after plasma donation. It confirmed that plasmapheresis method that used in convalescent plasma donation was a safe procedure.

## Limitation

The data that we have when this study is made is still minimal, which is influenced by several things, including the activities of convalescent plasma donors that have not been widely socialized and post-donor monitoring that is still limited. It is unfortunate that in this study the age stratification of plasma donors was less varied. This was due to the small number of survivors participating in this study, as well as the presence of several conditions in the older group of survivors that were unable to donate plasma, such as the need for a longer recovery period or certain comorbidities, therefore they cannot donate their plasma within the optimal timeframe for the required IgG or NAT levels. Rumors and stigma in society are also one of the reasons for the barrier. Therefore, further appropriate studies are required which involve more convalescent plasma donors that it could be used as a reference in educating the public regarding the advantages and disadvantages of convalescent plasma donor activities, both from the donor and recipient perspective.

## Conclusion

Immunological phenomenon in SARS-CoV-2, both in survivors and convalescent plasma donors, have not been widely observed and studied. From the case series discussed above, it can be concluded that convalescent plasma donation does not yet have strong evidence of decreasing levels of specific antibodies against SARS-CoV-2. There is a difference in immune response in survivors who have the same probability of being exposed to antigens with survivors who do not have this probability, where the group of survivors who are at risk of exposure to antigens after recovery can trigger anamnestic immune response that can increase antiSARS-CoV-2 IgG levels. Plasmapheresis procedure is safe to be done without reducing the protective effect of donor antibody post-plasma donation. More thorough prospective research is needed to prove this to increase the awareness of COVID-19 survivors to have higher willingness to donate convalescent plasma to accelerate the handling of cases of moderate-severe COVID-19 in this pandemic era.

## Data Availability Statement

The raw data supporting the conclusions of this article will be made available by the authors, without undue reservation.

## Ethics Statement

This study involved human participants. This were reviewed and approved by Ethics Committee of RSUD dr. Saiful Anwar, Malang, Indonesia; and also Commission for Health Research Ethics Research and Development Agency - Ministry of Health of Indonesia. The participants provided their written informed consent to participate in this study. Written informed consent was obtained from the individual(s) for the publication of any potentially identifiable images or data included in this article.

## Author Contributions

NA: collected the data of repetitive COVID-19 convalescence plasma donors in RSUD dr. Saiful Anwar Malang, Indonesia; collected the literature related to serial case reports; made the analysis and discussion of serial case reports, wrote the paper; other contribution: collected the COVID-19 convalescence plasma donors in RSUD dr. Saiful Anwar Malang, Indonesia. HB: collected the literature related to serial case reports; made the discussion of serial case reports; other contribution: collected the COVID-19 convalescence plasma donors in RSUD dr. Saiful Anwar Malang, Indonesia. SW: made the discussion of serial case reports; supervised the process in serial case reports making; other contribution: collected the COVID-19 convalescence plasma donors in RSUD dr. Saiful Anwar Malang, Indonesia. DH: made the discussion of serial case reports; supervised the process in serial case reports making. PA: made the discussion of serial case reports; supervised the process in serial case reports making. All authors contributed to the article and approved the submitted version.

## Conflict of Interest

The authors declare that the research was conducted in the absence of any commercial or financial relationships that could be construed as a potential conflict of interest.

## Publisher’s Note

All claims expressed in this article are solely those of the authors and do not necessarily represent those of their affiliated organizations, or those of the publisher, the editors and the reviewers. Any product that may be evaluated in this article, or claim that may be made by its manufacturer, is not guaranteed or endorsed by the publisher.
